# Three-year evaluation of different adhesion strategies in non-carious cervical lesion restorations: a randomized clinical trial*

**DOI:** 10.1590/1678-7757-2021-0192

**Published:** 2021-10-01

**Authors:** Diego Felipe Mardegan Gonçalves, Mirela Sanae Shinohara, Paulo Roberto Marão de Andrade Carvalho, Fernanda de Souza e Silva Ramos, Laryssa de Castro Oliveira, Érika Mayumi Omoto, Ticiane Cestari Fagundes

**Affiliations:** 1 Universidade Estadual Paulista Faculdade de Odontologia de Araçatuba Departamento de Odontologia Preventiva e Restauradora Araçatuba Brasil Universidade Estadual Paulista (UNESP), Faculdade de Odontologia de Araçatuba, Departamento de Odontologia Preventiva e Restauradora, Araçatuba, Brasil.; 2 Centro Universitário São Lucas Porto Velho Brasil Centro Universitário São Lucas, Porto Velho, Brasil.

**Keywords:** Acid etching, Adhesive systems, Non-carious cervical lesion, Glass-ionomer cements

## Abstract

**Objective:**

To evaluate non-carious cervical lesions (NCCLs) restored with different adhesion strategies.

**Methodology:**

This is a prospective, randomized, double-blind, split-mouth study. An adhesive restorative system (Single Bond Universal/Filtek Z350XT – SBU) was evaluated both without and with selective enamel conditioning (E-SBU), resin-modified glass-ionomer cements (Vitremer; RMGIC), and ethylenediaminetetraacetic acid pretreatment (EDTA; E-RMGIC). In total, 200 restorations, placed in 50 patients, were evaluated at baseline and at a 3-year follow-up using the modified United States Public Health Service (USPHS) criteria. Data were analyzed using the two-proportion equality test, multinomial logistic regression, Wilcoxon test, and Kaplan-Meier survival curves.

**Results:**

In total, 42 (84%) patients returned for the 3-year follow-up. SBU showed restoration losses statistically different from RMGIC. Retention was also statistically different in SBU between baseline and the 3-year follow-up. Marginal defects and surface texture were statistically significant for all groups in the period studied, except for the surface texture of SBU and the marginal integrity in E-RMGIC. We observed no statistically significant difference in wear, secondary caries, anatomical form, surface staining, and color over time. Recession degree was the only factor to influence retention rates. Cumulative survival (%) was 89, 98, 98, and 95.3, for SBU, SE-SBU, RMGIC, and E-RMGIC, respectively, without significant differences among them. There was a statistically significant difference between survival curves; however, multiple comparison procedures found no statistical differences.

**Conclusion:**

Selective enamel etching affected the retention of non-carious cervical restorations. Adhesion using EDTA and resin-modified glass-ionomer cements delayed marginal defects over time. The degree of gingival recession influenced retention rates. Resin composite restorations showed initial marginal defects, and ionomer restorations, reduced surface luster. EDTA pre-treatment followed by resin-modified glass-ionomer cements may be a promising adhesion strategy for NCCL restorations.

## Introduction

Non-carious cervical lesions (NCCL) are defined as the loss of tooth structure at the cemento-enamel junction area unrelated to dental caries, and their etiology has been described as multifactorial.^1^ Resin composites and glass-ionomer cements are currently the material of choice for NCCL restorations.^2^^–^^11^

Resin composites have some advantages, such as rapid polymerization, easy handling and reparability, and good mechanical and aesthetic properties;^12^ however, they also entail biological effects due to monomers release.^13^ This material peculiar features also pose challenges for its adhesion to the dentin substrate in NCCL.^14^ With the advent of universal adhesives, their composition enables the formation of chemical bonds, thus being less susceptible to hydrolytic degradation.^15^

Resin-modified glass-ionomer cements (RMGIC) can chemically bond to dental substrates. Since they can mechanically interlock with dentin, they are a good option for restoring NCCLs due to their excellent retention rates: between five and ten years.^16^^,^^17^ Given the excellent clinical retention of RMGIC - if applied under the manufacturers’ instructions, other pretreatment procedures may also be evaluated. The use of ethylenediaminetetraacetic acid (EDTA) before cement application is relevant, given that an *in vitro* study showed increased bond strength.^18^ Moreover, EDTA may be considered a metalloproteinases inhibitor (MMP).^19^

Though some clinical trials in the literature evaluate universal adhesives and RMGIC in NCCL restorations separetely,^2^^,^^3^ studies investigating these adhesion strategies in a same clinical trial after 3 years are scarce. Thus, our research aims to compare these adhesive treatments over time, and the influence of initial characteristics on NCCL restoration retention. The null hypotheses tested were: (1) the four adhesive strategies would present no statistically significant differences after 3 years, for each clinical criterion and survival analysis; (2) the same adhesive strategy would show no statistically significant difference between baseline and the 3-year follow-up; and (3) the initial characteristics of NCCLs would not influence restoration retention rates after 3 years.

## Methodology

### Study Design

This prospective, randomized, double-blind, split-mouth study aimed to evaluate the clinical performance of NCCL restorations using two restorative materials applied with different adhesion strategies. This study was conducted following the Consolidated Standards of Reporting Trials (CONSORT). The informed consent form used was analyzed and approved by the local Ethics Committee for investigations involving human subjects (#668.963). This study was registered on the Brazilian Clinical Trials Registry (RBR-655c3z). All participants provided written consent before treatment.

### Patient selection

Sample size was based on the retention rate of the Adper Single Bond (3M ESPE, St. Paul, USA), applied by simplified etch-and-rinse – the predecessor of the multi-mode adhesive. Its retention rate at 18 to 36-month follow-ups was reported to be 94%.^20^^–^^22^ Using 80% power, two-sided testing, the minimum sample size would have to be 50 restorations per group to detect a 20% group difference.

Fifty patients from the local undergraduate clinic were selected for this study, with at least four NCCLs each, regardless of their location in the dental arch.

Patients showing good health, no history of allergies to dental products and medications, and adequate oral hygiene without active periodontal disease were included in the study. Pregnant or lactating women, individuals with active carious lesions, users of desensitizing agents, fluorine or orthodontic appliances, or with severe bruxism with more than 50% wear were excluded.

### Teeth selection

Teeth with NCCL depth equal to or greater than 1 mm and a maximum depth of 4 mm, with at least 50% of their margins devoid of enamel were selected for the procedures. Careful clinical examination, including pulpal vitality tests (sensitivity to cold, hot, and percussion) verified the absence of periapical alterations in the selected teeth. No attempt was made to determine the etiology of the cervical lesions.

### Initial characteristics of teeth

Dentin features were evaluated according to their degree of sclerosis,^3^ as follows: 1 - no evident sclerosis, dentin is opaque, with yellow or whitish discoloration; 2- more than one but less than 50% sclerosis between categories 1 and 4; 3- less than 4, but more than 50% between categories 1 and 4; and 4- significant sclerosis present, dentin has a vitreous, dark yellow or even discolored (brown) appearance, with significant translucency or transparency.

Internal cavity angulation was measured and classified into 45-90°, 90-120°, or >120°. A millimeter probe measured the height, width, and depth of cavities in millimeters.

Gingival recession was categorized by the gingival margin in the mucogingival junction (MGJ) and the underlying alveolar bone as follows:^23^ class I - recession not extending to the MGJ, with no interdental bone or soft tissue loss; class II - recession extending to or beyond the MGJ, with no interdental bone or soft tissue loss; class III - recession extending to or beyond the MGJ, with loss of interdental bone or soft tissue apical to the cementoenamel junction (CEJ), but coronal to the apical extent of the recession; and class IV - recession extending to or beyond the MGJ, with interdental bone loss extending to a level apical to the recession.

Spontaneous sensitivity to air and probing were assessed via the visual analogue scale (VAS) for perceived pain^3^, thus scored: 1 - no pain; 2 - mild; 3 - moderate; 4 - slightly worse; 5 - much worse; and 6 - severe pain.

Procedure details can be found in our previous paper.^3^

### Randomization and restorative procedures

Prophylaxis with a pumice stone and water was performed before the restorative procedures. After color selection, initial photographs were taken; and anesthesia was applied locally when necessary. Relative isolation was performed using cotton rolls and suction; non-carious cervical lesions underwent no cavity preparation.

Two graduate students identified in the procedure sheet conducted the restorative procedures. One person unrelated to this study prepared opaque sealed envelopes identifying each group by their initials (employed to conceal the randomization sequence). Treatment was allocated to the groups, a tooth was raffled for one treatment, while the remaining were assigned other treatments, following the split-mouth design. It is important to emphasize that each operator performed the same number of restorative treatments, both for control and test groups.

For the SBU group, the adhesive system (Universal Single Bond - USB, 3M ESPE, St. Paul, USA) was applied to the NCCL under agitation for 20 s on a slightly dry surface followed by a 5-second light air jet. Then, the adhesive system was photocured for 10 s (Radii-cal, 1200 mW/cm^2^, SDI, Victoria, Australia).

For the E-SBU group, the enamel margin was conditioned with 37% phosphoric acid (Total Etch – Ivoclar Vivadent, Liechtenstein) for 15 s, washed with a water jet for 20 s, and slight dried. Then, the USB adhesive system was applied as described above.

Both groups were restored with a nanoparticulate resin composite (Filtek Z350XT, 3M ESPE, St. Paul, USA) applied in oblique increments. Each increment was photocured for 20 s via an exponential light technique, and the last increment, for 40 s. Fine-grained and extra-fine diamond tips, and sequential polishing discs were used for restoration finishing during the same appointment (Soflex Pop On, 3M ESPE, St. Paul, USA).

For the RMGIC group, RMGIC use (Vitremer, 3M ESPE, St. Paul, USA) followed the manufacturers’ instructions. After dental prophylaxis, the tooth surface was rinsed, dried; the primer was applied with a brush on NCCL surface, and photocured for 20 s. RMGICs were manipulated in a 1:1 powder/liquid volume ratio (powder portion to 1 drop of liquid) on a glass plate using a plastic spatula for approximately 45 s. After manipulation, the material was inserted into the cavity using a disposable tip attached to a Centrix syringe (DFL, Rio de Janeiro, Brazil). The restorative material was condensed against the cavity surfaces without the use of a matrix, and photocured for 40 s. Finishing and polishing used the method applied for resin composite restorations. Then, a thin protective coating was applied onto the surface and photocured for 20 s (Finishing Gloss, 3M ESPE St. Paul, USA).

For the E-RMGIC group, 0.1 M EDTA was applied onto the NCCL surface for 60 s with a brush. Then, the surface was washed with water for 30 s, dried, and restored with RMGIC. In this group, the primer was not applied on the lesion surface to allow the RMGIC to chemically bond to the dentin. The RMGIC were manipulated and inserted as described for Group III.

### Clinical evaluation

Restorations were evaluated by visual-tactile inspection using a flat mouth mirror, a periodontal probe, and a dental reflector; and classified according to the modified USPHS criteria. Restorations scored as Alpha or Bravo were considered clinically successful, those scored as Charlie, a failure. Two calibrated evaluators, blind to group assignment, examined and scored the restorations. In eventual disagreements, the evaluators reached a common agreement. Restorations were evaluated at baseline, and after 1, 2, and 3 years after the procedures.

### Statistical analysis

The two-proportions equality test was used to compare the four groups at each time-point and each group over time according to USPHS criteria. Multiple logistic regression analyses verified the influence of initial characteristics in restoration survival based on retention as a dependent variable. Cumulative survival was assessed by the Wilcoxon test, Kaplan-Meier survival curves, and Holm-Šidák pairwise comparisons. Significance level was set at 5%. All statistical analyses were performed in the SPSS version 20, and SigmaPlot version 13.

## Results

**Figure 1:**
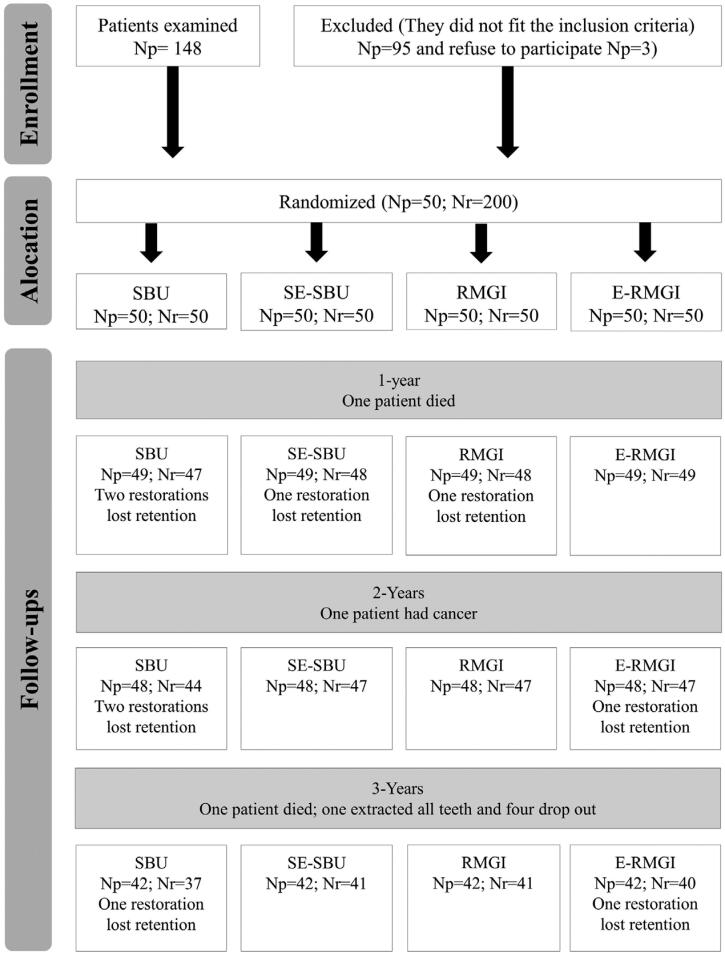
Patient flowchart. Np: patient number, Nr: restoration number

In total, 50 patients (34 male and 16 female) with a mean age of 61 years (ranging 38-92 years) participated in the study. We performed 200 restorations in total, homogenously distributed patients’ initial characteristics within four groups.^3^ Three years after the procedures, 42 (84%) patients returned for follow-up. Figure 1 shows restoration and patients’ justified absence at each follow-up. We performed no intention-to-treat analysis.

Table 1 shows clinical data. SBU contained five lost restorations statistically different to the RMGIC group, with only one (p≤0.05). We found no statistically significant differences among other criteria (p≥0.05). As for intra-comparisons, retention was statistically different in SBU from baseline to the 3-year follow-up (p≤0.05). Marginal defects and surface texture were likewise statistically different within all groups between the 3-year follow-up and baseline (p≤ 0.05), except for the surface texture of SBU and the marginal integrity of E-RMGIC (p≥0.05). The E-RMGIC group also showed gingival healing 3 years after the restorative procedure (p≤0.05). We verified no statistically significant difference for wear, secondary caries, anatomical form, surface staining, and color over time (p≥0.05).

**Table 1:** Numerical restoration results per group according to each criterion at baseline, and 1, 2 and 3 years after the procedure

Periods	Groups	Score	Retention	Marginal integrity	Marginal discoloration	Surface texture	Wear	Secondary caries	Anatomical form	Surface staining	Color	Gingival tissue
Baseline	SBU	A/B	50/-^Aa^	49/1^Aa^	50/- Aa	50/- Aa	50/- Aa	50/- Aa	49/1Aa	50/- Aa	36/14Aa	47/3Aa
		C	0	0	0	0	0	0	0	0	0	0
	SE-SBU	A/B	50/-^Aa^	49/1^Aa^	50/- Aa	50/- Aa	50/- Aa	50/- Aa	50/-Aa	50/- Aa	41/9Aa	47/3Aa
		C	0	0	0	0	0	0	0	0	0	0
	RMGI	A/B	50/-^Aa^	50/-^Aa^	50/- Aa	49/1 Aa	50/- Aa	50/- Aa	47/3Aa	50/- Aa	37/13Aa	47/3Aa
		C	0	0	0	0	0	0	0	0	0	0
	IV	A/B	50/-^Aa^	48/2^Aa^	50/- Aa	50/0Aa	50/- Aa	50/- Aa	48/2Aa	50/- Aa	39/11Aa	44/6Aa
		C	0	0	0	0	0	0	0	0	0	0
1 year	SBU	A/B	47/-^Aab^	38/9^Ab^	41/6Ab	45/2Aa	47/-Aa	47/-Aa	46/1Aa	45/2Aa	36/11^Aa^	46/1^Aa^
		C	2	0	0	0	0	0	0	0	0	0
	SE-SBU	A/B	48/-^Aa^	43/5^Aab^	43/5ABb	43/5Ab	48/-^Aa^	48/-^Aa^	48/-^Aa^	47/1^Aa^	38/10^Aa^	48/-^Aa^
		C	1	0	0	0	0	0	0	0	0	0
	RMGI	A/B	48/-^Aa^	44/4^Aab^	45/3A^Bab^	47/1^Aa^	48/-^Aa^	48/-^Aa^	46/2^Aa^	48/-^Aa^	33/15^Aa^	48/-^Aa^
		C	1	0	0	0	0	0	0	0	0	0
	E-RMGI	A/B	49/-^Aa^	44/5^Aa^	48/1^Ba^	47/2^Aab^	49/-^Aa^	49/-^Aa^	46/3^Aa^	49/0^Aa^	38/11^Aa^	49/-^Ab^
		C	0	0	0	0	0	0	0	0	0	0
2 years	SBU	A/B	44/-^Aa^	35/9^Ab^	35/9^Abc^	43/1^Aa^	44/-^Aa^	44/-^Aa^	43/1^Aa^	43/1^Aa^	36/8^Aa^	43/1^Aa^
		C	4	0	0	0	0	0	0	0	0	0
	SE-SBU	A/B	47/-^Aa^	40/7^Abc^	37/10^Abc^	43/4^Ab^	46/1^Aa^	47/-^Aa^	47/-^Aa^	45/2^Aa^	38/9^Aa^	47/-^Aa^
		C	1	0	0	0	0	0	0	0	0	0
	RMGI	A/B	47/-^Aa^	38/8^Abc^	42/5^Ab^	43/4^Aab^	46/1^Aa^	47/-^Aa^	45/2^Aa^	46/1^Aa^	33/14^Aa^	47/-^Aa^
		C	1	1	0	0	0	0	0	0	0	0
	E-RMGI	A/B	47/-^Aa^	37/10^Aa^	41/6^Ab^	42/5^Abc^	47/-^Aa^	47/-^Aa^	44/3^Aa^	47/-^Aa^	35/12^Aa^	47/-^Ab^
		C	1	0	0	0	0	0	0	0	0	0
3 years	SBU	A/B	37/-^Bb^	25/12^Ab^	25/12^Ac^	35/2^Aa^	36/1^Aa^	37/-^Aa^	36/1^Aa^	35/2^Aa^	29/8^Aa^	36/1Aa
		C	5	0	0	0	0	0	0	0	0	0
	SE-SBU	A/B	41/-^ABa^	30/11^Ac^	25/16^Ac^	36/5^Ab^	38/3^Aa^	41/-^Aa^	41/-^Aa^	39/2^Aa^	32/9^Aa^	41/-^Aa^
		C	1	0	0	0	0	0	0	0	0	0
	RMGI	A/B	42/-^Aa^	30/11^Ac^	29/13^Ac^	34/8^Ab^	39/3^Aa^	42/-^Aa^	40/2^Aa^	40/2^Aa^	27/15^Aa^	42/-^Aa^
		C	1	1	0	0	0	0	0	0	0	0
	E-RMGI	A/B	40/-^ABa^	30/10^Aa^	30/10^Ab^	32/8^Ac^	38/2^Aa^	40/-^Aa^	38/2^Aa^	40/-^Aa^	32/8^Aa^	40/-^Ab^
		C	2	0	0	0	0	0	0	0	0	0

Distinct uppercase letters compare groups by the same evaluation; distinct lowercase letters compare the same group over time.

**Figure 2:**
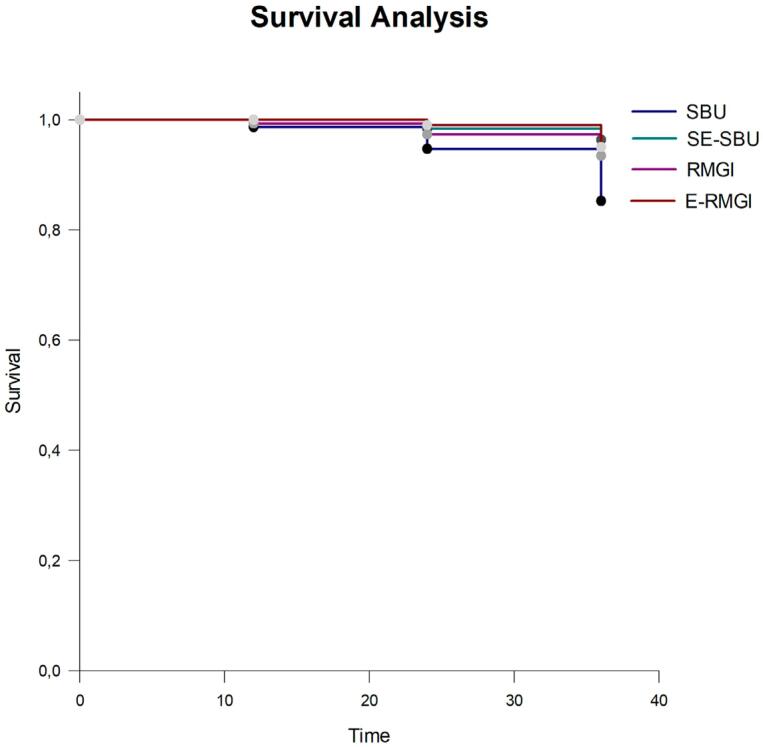
Retention survival comparison among the four groups

The 3-year cumulative survival (%) was 89, 98, 98, and 95.3, for SBU, SE-SBU, RMGIC, and E-RMGIC, respectively, without significant differences among them. Figure 2 shows the statistically significant difference between survival curves (p=0.045); however, multiple comparisons found no statistical differences (p≥0.05). The degree of gingival recession was the only initial characteristic to influence restoration retention (Table 2).

**Table 2:** NCCL initial characteristics per group

NCCL characteristics	Groups					p value
	SBU	SE-SBU	RMGI	E-RMGI	Total	
**Tooth distribution**						
**Incisor**	6	7	7	5 (1)	25	0.771
**Canine**	6 (1)	4	7	8	25	
**Premolar**	29 (2)	33 (1)	32 (1)	31 (1)	125	
**Molar**	9 (2)	6	4	6	25	
**Degree of sclerosis**						
**1**	18 (2)	16	13	18	65	0.201
**2**	15	21 (1)	22 (1)	21 (1)	79	
**3**	15 (2)	9	11	8 (1)	43	
**4**	2 (1)	4	4	3	13	
**Internal angles**						
**45-90º**	11 (2)	14 (1)	12	13 (2)	50	0.488
**90-120º**	12 (1)	16	16	14	58	
**>120º**	27 (2)	20	22 (1)	23	92	
**Cervico-incisal height (mm)**						
**<1.5**	0	4	6	5 (1)	15	0.351
**1.5-2.5**	17 (1)	20 (1)	13 (1)	22 (1)	72	
**2.5-4.0**	25 (3)	22	26	16	89	
**>4.0**	8 (1)	4	6	6	24	
**Width (mm)**						
**1-2**	5	5	8	7 (1)	25	0.430
**3-4**	34 (3)	35 (1)	36 (1)	38 (1)	143	
**5-6**	7 (1)	7	5	4	23	
**7-8**	4 (1)	3	1	1	9	
**Depth (mm)**						
**1-2**	48 (3)	48 (1)	48 (1)	50 (2)	194	0.953
**3-4**	2 (2)	2	2	–	6	
**Degree of gingival recession**						
**1**	42 (3)	40	40 (1)	41 (1)	163	0.045
**2**	7 (1)	10 (1)	10	8 (1)	35	
**3**	1 (1)	1	0	0	2	
**Pre-operative pain (air dry)**						
**1**	28 (3)	27	30 (1)	28 (2)	113	0.804
**2**	9	11 (1)	8	8	36	
**3**	5	4	3	4	16	
**4**	4 (2)	3	3	3	13	
**5**	3	4	5	5	17	
**6**	0	0	0	0	0	
**Pre-operative pain (spontaneous)**						
**1**	46 (4)	47 (1)	48 (1)	48 (2)	189	0.982
**2**	1 (1)	0	1	1	3	
**3**	2	1	0	0	3	
**4**	1	1	1	1	4	
**5**	0	1	0	0	1	
**6**	0	0	0	0	0	

Multiple logistic regression was used as statistical test. Parentheses show retention failures.

## Discussion

Several clinical studies in the literature address non-carious cervical lesions (NCCLs). In restoring these lesions, the chemical interaction with the dental structure is important for the quality and durability of adhesion.^24^ Adhesion efficacy improved after the introduction of multi-mode adhesives containing 10-Methacryloyloxydecyl dihydrogen phosphate (10-MDP) which interacts chemically with dental substrates.^25^ Glass-ionomer materials promoted retentive stability due to their chemical interactions with the dentin substrate found in NCCL.^25^ Moreover, glass-ionomer cements delayed the acid-induced dissolution rate of root dentin.^26^ A systematic review and meta-analysis found a superior retention rate in glass-ionomer restorations than resin composite ones; though, other parameters showed no significant difference.^4^

Retention is one of the most important evaluating criteria for restorative material performance, often used to assess its longevity. The American Dental Association (ADA) guidelines on adhesive materials requires the cumulative 18-month retention rate to be at least 90% for procedures in dentin and enamel to be fully acceptable.^27^ However, the guidelines have no requirements for the long-term durability of adhesive systems. In our study, SBU lost approximately 11.9% retention three years after the procedure – the highest value recorded, – thus rejecting the first null hypothesis. Meta-analysis showed universal adhesives to have better adhesion after enamel etching.^28^ In that sense, universal adhesives applied through etch-and-rinse and selective-etch modes tend to achieve better clinical outcomes.^2^^,^^5^^,^^6^^,^^8^ In our study, the group without selective enamel conditioning presented high initial debonding rates, and SBU, the lowest 3-year cumulative survival (89%). Some clinical studies evaluating universal adhesive restorations, based on USPHS or World Dental Federation (*Fédération Dentaire Internationale* - FDI) criteria, and 6 to 36-month follow-ups, also reported few clinical changes over time.^2^^–^^10^

The RMGIC group showed a cumulative survival retention (98%) similar to other clinical trials assessing the same ionomer brand (Vitremer) after 5 years (93-96.4%).^17^^,^^29^ According to Luque-Martinez, et al. (2015), EDTA conditioning improves self-etching adhesive restoration retention rates 18 months after the procedures.^30^ However, the literature lacks a clinical study evaluating the use of EDTA prior to the application of ionomeric cements in NCCLs, thus hindering a comparison with our results. Acting in zinc and calcium ions essential for metalloproteinase (MMPs) activity, EDTA may be an alternative pretreatment for NCCLs restorations,^31^ possibly explaining the retention of all restorations in the E-RMGIC group at their 1-year follow-up.

When reliable adhesives are properly used, early restoration loss may not be the main clinical problem, given that marginal discoloration increases over time and may become a more prominent reason for repair or replacement.^32^ At the 3-year follow-up, RMGIC restorations reached more acceptable ratings for marginal discoloration than resin composite restorations, thus rejecting our second hypothesis. Approximately 70% of NCCLs restored with RMGIC obtained Alpha scores for marginal discoloration, corroborating the literature results, which verified an Alpha score for this criterion ranging from 42.9% to 84.6% after 5 years of the procedure.^29^^,^^33^

Studies found restorations that were minimum 2-3 years old *in vivo* to present an enamel-like layer adjacent to the ionomer due to a calcium and phosphorus increase in this surface layer. This suggests an additional “mineralization” of the restorative material,^34^^,^^35^ possibly contributing to its best performance as to its marginal defects. The E-RMGIC group showed no statistical differences in marginal integrity between baseline and the 3-year follow-up, and marginal discoloration over time was delayed in this group. EDTA obtained less microleakage in primary teeth compared to other pretreatment alternatives for restorations with glass-ionomer cements, thus constituting an alternative to promote chemical and micromechanical adhesion of this material to the dental structure.^36^

However, higher surface roughness was observed in ionomer restorations, corroborating the literature results.^37^ In our study, approximately 80% of ionomer restorations reached an Alpha score for surface texture. The literature reports a progressive increase in surface roughness of ionomer restorations after 5 years, with Alpha scores ranging from 21.4% to 23%.^29^^,^^33^ Gingival tissue scores improved after three years of the procedure along with an improvement in patients’ dental hygiene, which may be owed to the referral for additional treatments in undergraduate clinics.

Multiple logistic regression showed how gingival recession influenced restoration retention after 3 years, also rejecting our third null hypothesis. Considering the multifactorial etiology of NCCLs, oral health, teeth positioning in the dental arch, and occlusal interferences and overloads may influence the apical migration of the gingival margin over the years.^38^ Nine restorations were lost between follow-ups, whereby five were classified as degree 1, three as degree 2, and one as degree 3. Other factors that may have contributed for retention failures are the exposure of restoration margins (mainly in the SBU group, lacking enamel conditioning), the effect of occlusal forces, or excessive pressure during brushing.^39^

NCCLs have a multifactorial etiology, including erosion (chemical degradation), mechanical stress (tension), and friction.^40^ It is known that risk factors such as occlusal forces, overbrushing, and acidic beverages may influence the evolution in NCLL intensity, duration, and frequency.^40^ Our clinical trial faced different challenges in controlling all influencing factors, and providing enough evidence to conclusively support the causes of the lesions,^41^ we, thus, did not classify the type of NCCL – which may be deemed a limitation of this study.^41^ Furthermore, attrition may have introduced biases because the characteristics of individuals absent for follow-ups can differ between the randomized groups.^42^ Longer follow-up periods would most likely detect more differences among adhesion strategies for NCCL restorations.

## Conclusions

Based on the 3-year results of this randomized clinical trial, we conclude that selective enamel etching affected NCCL restoration retention. Furthermore, the adhesive strategy using EDTA followed by RMGIC delayed marginal defects over time. Finally, the degree of gingival recession may affect NCCL restoration retention.
